# Dynamic Activation and Repression of the *Plasmodium falciparum rif* Gene Family and Their Relation to Chromatin Modification

**DOI:** 10.1371/journal.pone.0029881

**Published:** 2012-01-03

**Authors:** Fernanda J. Cabral, Wesley L. Fotoran, Gerhard Wunderlich

**Affiliations:** Department of Parasitology, Institute of Biomedical Sciences, University of São Paulo, São Paulo, São Paulo, Brazil; Federal University of São Paulo, Brazil

## Abstract

The regulation of variant gene expression in *Plasmodium falciparum* is still only partially understood. Regulation of *var* genes, the most studied gene family involved in antigenic variation, is orchestrated by a dynamic pattern of inherited chromatin states. Although recent evidence pointed to epigenetic regulation of transcribed and repressed *rif* loci, little is known about specific on/off associated histone modifications of individual *rif* genes. To investigate the chromatin marks for transcribed and repressed *rif* loci, we cultivated parasites and evaluated the transcriptional status of chosen *rif* targets by qRT-PCR and performed ChIP assays using H3K9ac and H3K9me3 antibodies. We then monitored changes in the epigenetic patterns in parasites after several reinvasions and also evaluated the “poised” mark in trophozoites and schizonts of the same erythrocytic cycle by ChIP using H3K4me2 specific antibodies. Our results show that H3K9 is acetylated in transcribed *rif* loci and trimethylated or even unmodified in repressed *rif* loci. These transcriptional and epigenetic states are inherited after several reinvasions. The poised modification H3K4me2 showed a tendency to be more present in loci in trophozoites that upon progression to schizonts strongly transcribe the respective locus. However, this effect was not consistently observed for all monitored loci. While our data show important similarities to *var* transcription-associated chromatin modifications, the observed swiftly occurring modifications at *rif* loci and the absence of H3K9 modification point to a different dynamic of recruitment of chromatin modifying enzymes.

## Introduction

The human malaria parasite *Plasmodium falciparum* maintains its persistence in the vertebrate host at least in part by expression of several variant proteins at the surface of infected erythrocytes. The most important variant antigen is PfEMP-1 (*Plasmodium falciparum* erythrocyte membrane protein 1) which is encoded by approximately 60 members of the *var* multigene family [1]. The transcription of this gene family is strictly controlled by a complex mechanism termed allelic exclusion and it is assumed that one or very few *var* genes are expressed per single parasite [2]. Occasional transcriptional switching which occurs at rates ranging from 0.2 up to 16% then leads to the change in the expressed PfEMP-1 [3], [4], enabling constant immune evasion. While the purpose of PfEMP-1 is the interaction with host cell receptors leading to the retention of the infected red blood cell in the deep vasculature thus avoiding spleen passage, no clear function has so far been elucidated for proteins encoded by other multigene families. Proteins of RIFIN, STEVOR and PFM2tm families are also expressed at the infected red blood cell surface and little is known about the function of these antigens regarding malaria persistence, pathogeny and cytoadherence.

The multigene *rif* family has approximately 160 different intact members in the 3D7 strain annotated in the PlasmoDB databank. Recently, we [5] and others [6] have characterized the transcriptional profile of this family, showing that i) particular, sometimes identical transcripts are expressed in different adhesive phenotypes and ii) that *rif* transcription switches apparently faster than transcription of *var* genes, at least *in vitro*. Similar to *var* genes, *rif* gene transcription and silencing is dependent on the histone deacetylase PfSir2A [7] and genome-wide chromatin immunoprecipitation experiments have shown that silent *rif* gene loci are associated to histone 3 lysine 9 trimethylation as are other virulence-associated multigene families [8], [9]. Finally, results from the laboratory of Kirk Deitsch have shown that the transcription of several variant multigene families, namely *var*, *rif*, and *stevor* seem to be controlled by a common factor and overexpression of artificial loci containing a *rif* promoter suppressed the transcription of *var* loci [10]. The hypothesis of a recruitment of variant gene promoters to a specific, physically limited site in the nucleus, which may explain the observed allelic exclusion at least for *var* genes, recently gained further support [10], [11].

At the molecular level, Lopez-Rubio and colleagues [12] have shown that a *var* gene which is actively transcribed was associated to histones carrying an acetyl group at the lysine 9 of histone 3 (H3K9) and Chookajorn and colleagues [13] have shown that the same silenced locus was associated to trimethylated lysine 9 at histone 3. Additionally, Lopez-Rubio and colleagues have shown that a *var* locus active in early trophozoite stage and silenced in schizont stage (“poised” state, to be transcribed after the following reinvasion) was associated with a dimethylated lysine 4 in histone 3 [12].

Given the observation of similar histone landmarks in ChIP-on-Chip experiments [9] and the probable existence of a common factor controlling transcription of multigene families in *P. falciparum*, we addressed the question if dynamic histone modifications which are decisive in *var* regulation are also associated with active or silent *rif* loci. For this, the transcription of *rif* genes was monitored in consecutive cultures and compared to the histone modifications found for each locus in the same parasites.

## Results

### Genomic loci containing active *rif* genes are preferentially acetylated and repressed loci are trimethylated or unmodified at H3K9

**Figure 1:**
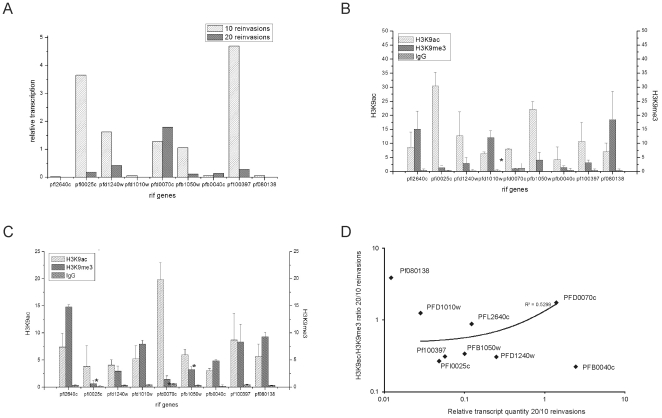
Transcriptional analysis and chromatin enrichment after 10 and 20 reinvasions. A: Transcription of 3D7-CD36 parasites of chosen *rif* targets are shown as relative values (2^−ΔCt^) using the gene of plasmodial t-seryl Synthetase (PF070073) as an internal control. B, C: Chromatin enrichment at selected *rif* loci shown as H3K9ac/H3K9me3 ratios obtained for 20–24 h trophozoite samples after 10 (B) and 20 reinvasions (C). D: Scatter plot showing the relation of histone modifications and transcript quantity. The coefficients of relative transcription at each loci (at 20 and 10 reinvasions) were plotted against the coefficient of H3K9ac/me3 ratios_20 reinvasions_/H3K9ac/me3 ratios_10 reinvasions_. The ChIP experiment was done in biological triplicates and Pearson's correlation of the values is indicated (p = 0.03, one tailed probability). Note that the extreme outliers PF080138 and PFB0040c were not considered.

*Var* gene loci in their active state are associated with class 3 histones (H3) carrying an acetylation mark at their lysine 9 (H3K9ac), while silenced *var* loci associate with H3K9me3 [12]. In order to establish if the dynamics of activation and repression of *rif* loci are associated with the same epigenetic landmarks as *var* loci, we first performed a qRT-PCR-based *rif* transcript analysis in parasites previously selected for cytoadherence in CHO-CD36, since in this phenotype a number of *rif* loci were found active with rapid switching after a small number of reinvasions and given the large number of *rif* genes the analysis was restricted to these loci [5]. We monitored the transcriptional status of this set of *rif* genes after 10 and 20 reinvasions after the last panning procedure. As expected, a number of *rif* genes were actively transcribed while others were silenced or transcribed at background levels (<0.1 relative transcription units (Figure 1A). The pattern of transcription changed swiftly after 20 reinvasions: While in parasites 10 reinvasions after cytoadherence selection PFI0025c, PFD1240w, PFD0070c PFB1050w and PF100397 were still significantly transcribed (>0.1 relative transcription units), other tested transcripts were barely detectable (Figure 1A). With the exception of PFD0070c, all tested transcripts decreased further after 20 reinvasions. In order to evaluate the epigenetic state and the posttranslational modifications of the chromatin associated to the monitored *rif* loci, ChIP was performed using H3K9me3 and H3K9ac-specific antibodies. These antibodies were previously used in ChIP experiments examining *var* gene expression and it was established that H3K9me3 and H3K9ac are epigenetic marks for repressed and active *var* loci, respectively [12]. Unrelated IgG immunoprecipitated nucleosomes almost at background levels in all of the experiments which demonstrated the specificity of the antibodies and immunoprecipitation assays (Figure 1 B and C). Due to the impossibility of designing specific, non-ambiguous PCR primers for *rif* 5′-upstream regions, the same oligonucleotides as used for the transcription analysis were employed for the detection of chromatin associated *rif* sequences (for primer localization and genomic context of *rif* targets see Figure S2).

In general, we observed that *rif* genes which showed high transcript quantities were associated dominantly with acetylated H3K9 (Figure S1). Specifically, in samples from the 10^th^ reinvasion, the loci PFI0025c and PF100397 were the most transcribed and they were preferentially acetylated (Figure 1B), however, only three loci showed statistically significant differences comparing the 10-reinvasion sample with the 20-reinvasion sample (PFD0070c, PFI0025c, PFB1050w). In contrast, silent *rif* loci PFL2640c, PFD1010w and PF080138 seemed preferentially methylated, coinciding with the previous epigenome results [8], [9]. After 20 reinvasions, most of the loci showed only low transcript quantities with the exception of PFD0070c which showed almost 5 times more transcript than the internal control used for calibration. Coincidently, the acetylation was strongly and significantly increased at this locus (Fig. 1C). The remaining tested *rif* transcripts were barely detectable (<0.5 relative units). Noteworthy, the overall intensity of immunoprecipitation varied between loci. In order to show if predominant H3K9-acetylation - expressed as a higher ratio H3K9ac/H3K9me3 – statistically correlated with higher transcript abundance, we divided the H3K9ac/H3K9me3 ratios after 20 reinvasions by the H3K9ac/H3K9me3 ratios after 10 reinvasions and plotted them against the coefficients of relative transcript quantity from each locus after 20 and 10 reinvasions (Figure 1D). If H3K9ac correlates with higher transcription, then the plotted data points should show a positive correlation, which is indeed observed (p = 0.03, R^2^ = 0.5299). The extreme outliers were not considered in the analysis (Pf080138 and PFB0040c). A significant correlation of H3K9ac/H3K9me3 and transcript quantities was also observed when the data were analyzed separately for the 10 and 20 reinvasion material (Figure S1).

**Figure 2 pone-0029881-g002:**
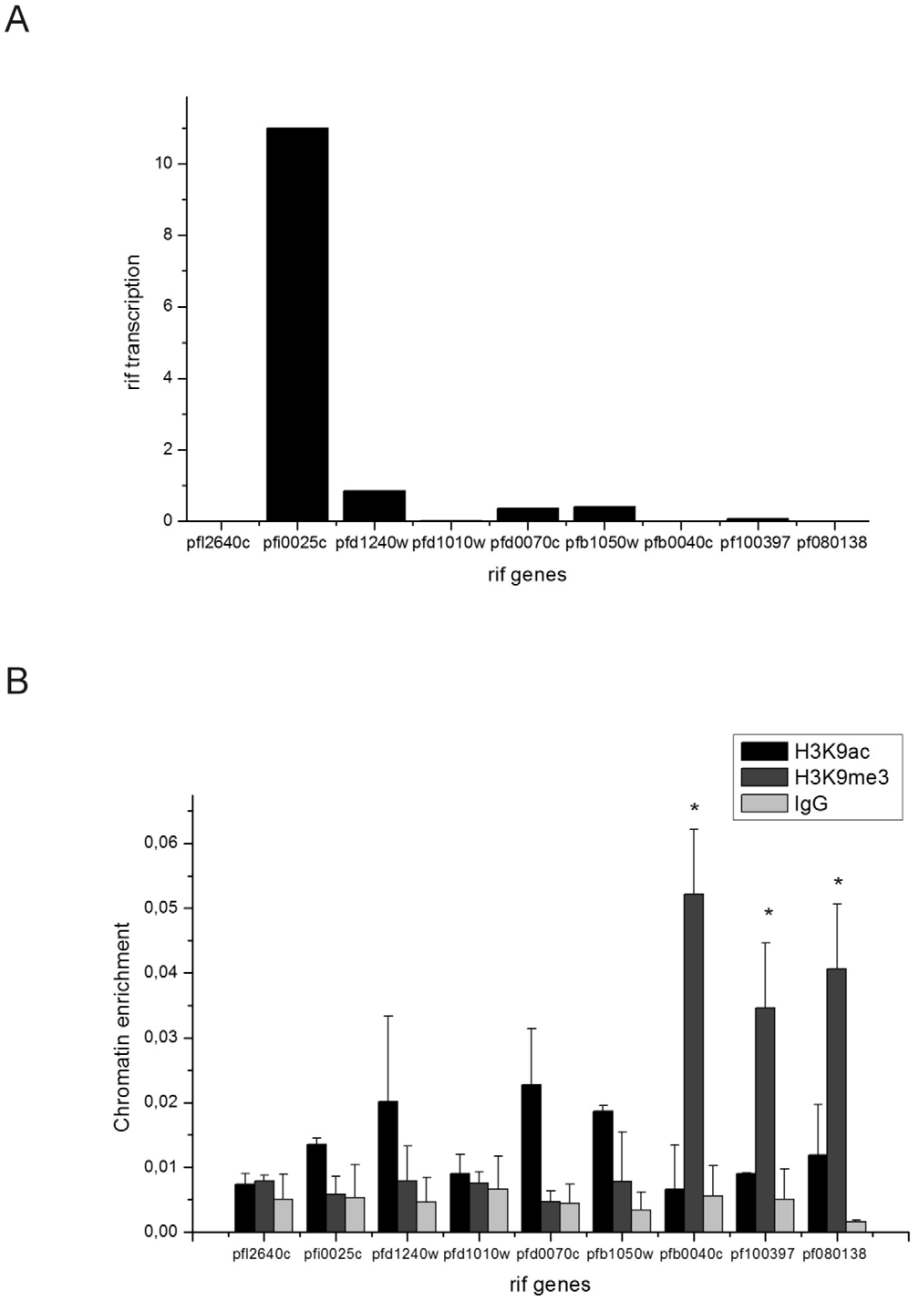
*Rif* transcription and H3K9 modifications after reselection over CHO-CD36 cells. (A) The *rif* transcript quantities of freshly CHO-CD36 selected parasites in trophozoite stage (20–24 h post invasion) were monitored by RT-qPCR in relation to the internal control PF070073. (B) Histone modifications were measured by ChIP as described and significant differences in signals between H3K9ac and H3K9me3 modifications are depicted by asterisks (Student's T test, *p*<0.05). ChIP was performed in triplicates.

We then asked what happened to *rif* transcription and loci modification if the outgrown parasites (after 20 reinvasions) were reselected for cytoadherence on CHO-CD36 cells. After two selection rounds, trophozoites at 20–24 h post reinvasion showed most transcripts from the PFI0025c locus and minor quantities from loci PFD1240w, PFD0070c and PF100397 (Figure 2). As before, transcribed loci showed higher H3K9ac/H3K9me3 ratios, while loci which showed few transcripts (<0.2 relative units) showed either higher H3K9 methylation marks or the absence of any specific mark. Importantly, the apparently unmodified and transcriptionally silent loci PFL2640c and PFD1010w were similarly immunoprecipitated by antiH3 as the silent but H3K9-trimethylated loci PFB0040c and PF080138 (data not shown). When looking for *var* transcription in this parasite line, we observed that the dominant *var* transcript was PFD0615c, as previously described [14] (data not shown). Notably, another lot of antiH3, antiH3K9me3 and antiH3K9ac were employed in this experiment and these showed different immunoprecipitation efficiencies, explaining the altered recovery of acetylated and trimethylated histones 3 when compared to the experiment shown in Figure 1.

### Stronger dimethylation at H3K4 seem to associate with transcription/repression at active or silent loci

**Figure 3:**
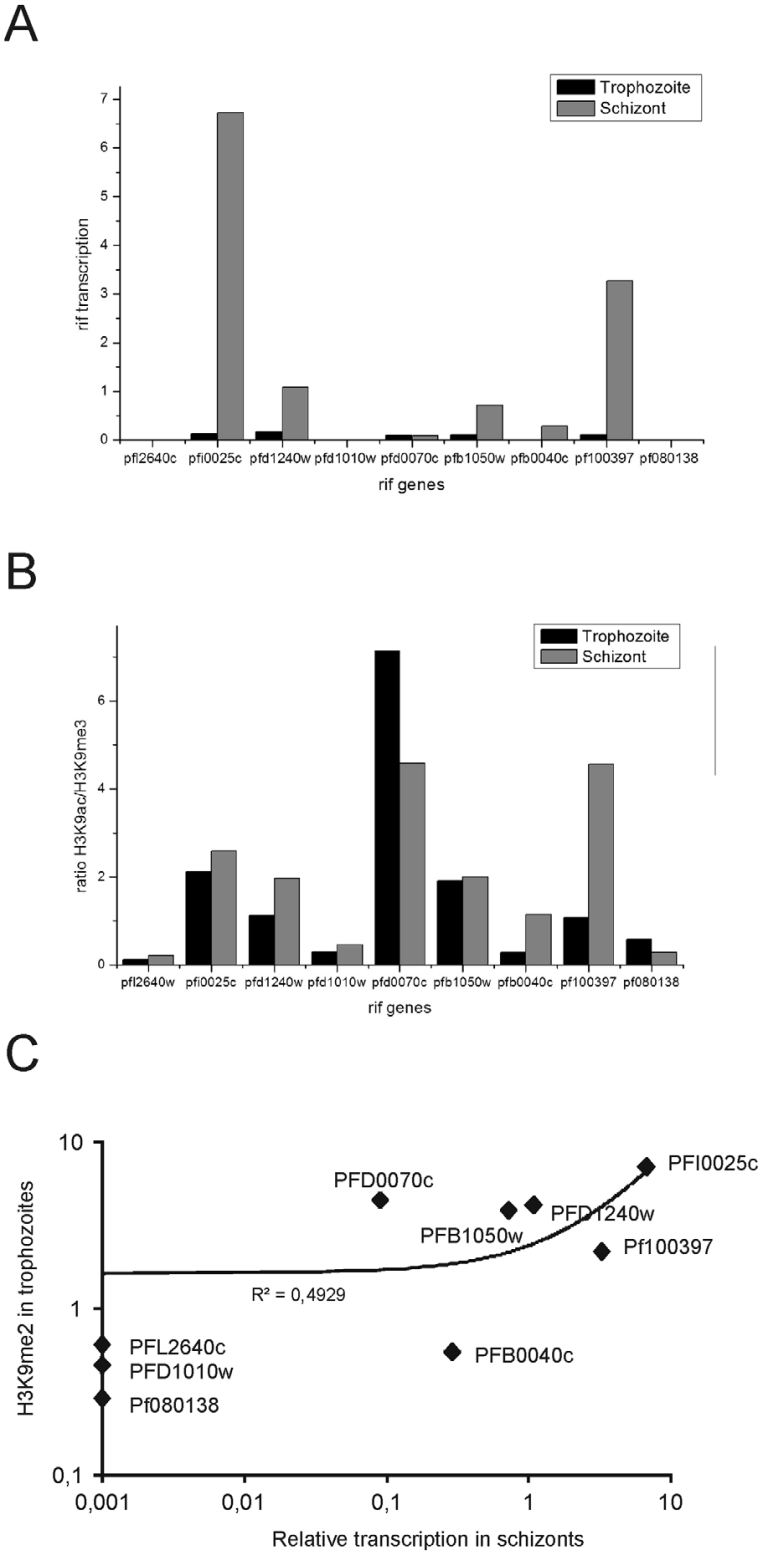
Relative quantities of *rif* transcripts and chromatin enrichment for H3K9ac, H3K9me3 and H3K4me2 (poised mark) in trophozoite and schizont stages for selected *rif* loci. A: Relative transcript amounts of chosen *rif* targets in trophozoites and schizonts in 3D7 parasites of the same reinvasion, calculated as above. B: Ratios of H3K9ac/me3 enrichment in trophozoites (20–24 h p.i.) and schizonts (30–34 h p.i.). C: Scatter plots of the relative transcription in schizonts plotted against the H3K4me2 enrichment in trophozoites showing a positive and significant correlation (R^2^ = 0.4929, p = 0.02). The ChIP experiment was done in biological triplicates.

Transcription of *var* genes occurs normally in a conserved manner and the transcriptional switching is relatively slow, at least in *in vitro* cultures. In schizonts, where all *var* genes are transcriptionally silent, a *var* locus that is transcribed after the next reinvasion is marked at H3K4 with a dimethyl moiety (H3K4me2) [12]. In order to evaluate if the same modification is found for *rif* loci which are actively transcribed in trophozoites (20–24 h post reinvasion) and/or schizonts (30–34 h post reinvasion), we cultivated parasites previously cytoadherence-selected on CHO-CD36 (25 reinvasions after selection), synchronized them and harvested chromatin 20 and 30 h post reinvasion and analyzed for their modification at H3K9 and H3K4 by ChIP. In trophozoites, we detected relatively low *rif* transcription at the tested loci (below 0.5 relative units), while in schizonts more transcripts accumulated (Figure 3A). After ChIP analysis, *rif* loci showed a tendency to slightly increased acetylation over trimethylation when transcripts were detected (above 0.1 relative units) and there was a significant correlation of transcript quantity with H3K9ac/H3K9me3 ratios in schizonts but not in trophozoites (Figure S1). With the exception of PFD0070c, stronger trimethylation than acetylation was always associated with the complete absence of transcripts (Fig. 3A, B) and this was also true for schizonts. For example, the transcribed *rif* PFB0040c (silent in trophozoites) showed increased acetylation at H3K9 when compared to trophozoites, and the strongly transcribed PF100397 showed accentuated acetylation. The only locus which did not follow the pattern was PFD0070c, which showed very few transcripts in trophozoites and in schizonts, although its locus was strongly acetylated at H3K9. The loci with no detectable transcription in trophozoites or in schizonts followed the pattern of either stronger trimethylation or lower levels of any modification (Figure S3).

The transcripts of genes PFI0025c, PFD1240w, PFB1050w, PFB0040c and PF100397 increased in schizonts compared to trophozoites. Therefore, we asked if the “poised mark” H3K4me2 [12] in trophozoites correlated with relative transcript quantities in schizonts. A positive correlation was observed (R^2^ = 0.49, p = 0.017) when we plotted the transcript quantities against the H3K4me2/H3 enrichment ratio. The silent loci PFL2640, PFD1010w, PF080138 and the weakly transcribed locus PFB0040c were not significantly marked (Figure 3 C and Figure S3 B and C). Again, the locus PFD0070c behaved in an exceptional manner showing significant H3K4me2 modification together with a high H3K9ac/me3 ratio but without significant transcription. Taken together, it appears that the dimethylation at H3K4 in trophozoites is correlated to active transcription in the later schizont stages but this connection is not so clearcut as for poised *var* genes.

## Discussion

Posttranslational modification (PTM) of histones is a well-known mechanism that is related to the control of gene expression in eukaryotes. It is believed that a “histone code” orchestrates the accessibility of transcription factors to promoter regions through modification of histone tails by methyl, acetyl and phosphate groups, and proteins as ubiquitin and SUMO as reviewed by Berger [15]. A number of these modifications were also found in *P. falciparum* [16]. The histone code is modulated by proteins such as histone methylases, de-methylases and other transcriptional cofactors such as Polycomb proteins, that play a role as regulators of the activation or repression of gene expression [17], [18]. In *P. falciparum* several efforts are directed to elucidate the role of epigenetic events in the control of transcription and antigenic variation. Recently, a study involving ChIP-on-chip showed that clonally variant genes are highly enriched in H3K9me3 at heterochromatin sites [8] and are restricted to subtelomeric regions and islands in central positions of a number of chromosomes [9], however, this work did not correlate individual histone modifications with transcriptional activity at determined *rif* loci. Also, a specific common factor was postulated to control gene expression of clonally variant families [10] which may directly interact with the chromatin. In addition, it was established that antigenic variation and allelic exclusion is maintained by heterochromatin formation and intranuclear repositioning of *var* loci [19]. Another study showed that the nucleosomal occupancy differed greatly between loci of *var* genes in dependence of the position of the gene (higher occupancy at the exon 2 region in the 3′ part of the coding region) [20].

In order to elucidate mechanisms responsible for *rif* gene transcription activation and silencing, we performed a detailed analysis of a number of *rif* genes. Since the selective upregulation of *rif* genes by phenotypic selection is not yet possible as it is for *var* genes e.g. [21], [14], we took advantage of the accelerated switching of *rif* genes and the frequent transcriptional activity of the herein tested loci [5] and monitored parasite cultures over 10 reinvasion cycles. Over the time, “on” and “off” states were observed in a number of loci and permitted the measurement of the underlying histone modifications.

**Figure 4:**
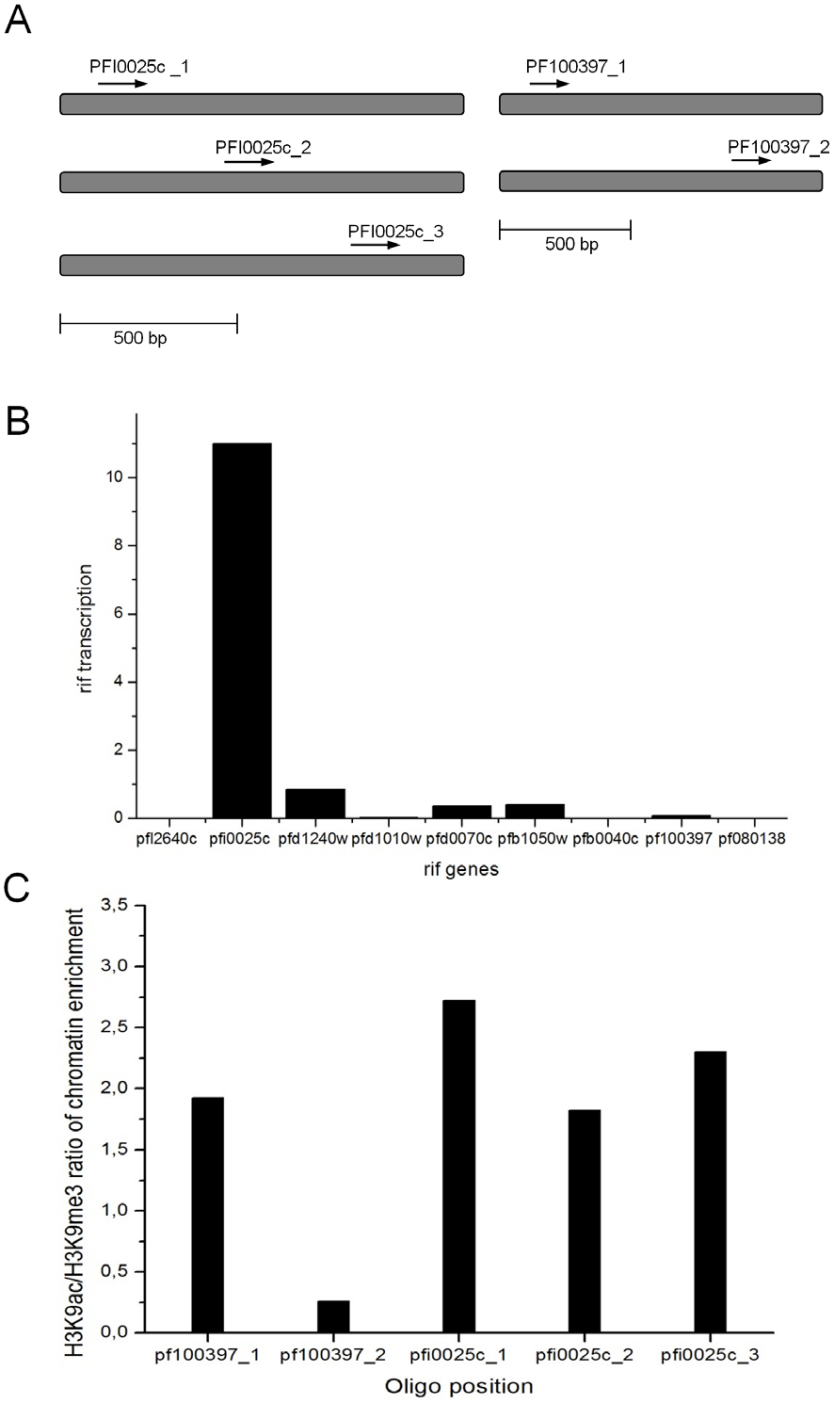
Influence of primer localization on the qPCR signal after ChIP at two *rif* loci. A: Immunoprecipitated material from the experiment in Fig. 2 was used and the localization of the employed primer pairs in qPCR is shown for the two tested *rif* ORFs. B: *rif* transcription at the two loci was measured as in Figure 1. C: The H3K9ac and H3K9me3 signals in ChIP were expressed as ratios. Note that the oligonucleotide primers used for the experiments in Figures 1–3 were PFI0025c_3 and PF100397_2. The ChIP experiment was done in biological triplicates.

Our results show that modifications which control *var* gene locus activity seem at least partially also functional for *rif* loci: When a significant presence of transcripts for any tested locus was observed, H3K9 acetylation dominated. This mark has not been identified before for active *rif* genes. Inversely, in the absence of transcripts, loci were either associated with strong tri-methylation of H3K9, in accordance to data published by Lopez-Rubio and colleagues [12], or remained almost unmodified. As previously shown for *var* loci, the modifications are dynamic [13] and change over time, mostly in relation to the observed transcript quantities. The intensity of methylation or acetylation – reflected as stronger or weaker amplification in ChIP assays – seemed to vary between different loci. At least two reasons may account for this observation: Firstly, the localization of oligos used for qPCR were different in each gene: If the PTM at H3 follow the pattern of *var* genes, one would perhaps expect higher acetylation in “on” *rif* genes of which oligos amplify in the 5′ region of the gene – in proximity to the promoter region which is supposed to carry most of the acetyl residues [12]. However, *rif* genes are much shorter than the sometimes 12 kb-*var* genes. It is possible that the short central 500 bp range in which the oligos were allocated in the different *rif* genes may not allow to discriminate these positional effects, taking also in account that the immunoprecipitated genomic DNA fragments generated during the ChIP procedure had around 400 bp (data not shown). In order to address the question of positional effects of priming sites in genes analyzed by ChIP, we compared signal differences in the loci PFI0025c and PF100397, using additional specific oligo pairs in the coding sequence. For this experiment, material from the experiment in Figure 2 was used, and in these parasites locus PFI0025c was transcriptionally active while PF100397 showed only few transcripts. Although statistically not distinguishable, acetylation and to a lower degree methylation seem more present in the 5′ region of the coding sequence when transcription is active (Figure 4, Figure S4 A), while methylation is increased in the 3′ region of the open reading frame of a weakly transcribed *rif* gene (Figure 4 B and C, Figure S4 B). If these findings are extrapolated to the other loci measured herein, it may be argued that the H3K9me3 values found for silent loci with oligonucleotides priming in the 5′ region are underestimated. On the other hand, active loci appear to be modified almost uniformly throughout the coding sequence as shown by almost equal H3K9ac/me3 ratios (Figure 4 C).

The second reason for disparate methylation/acetylation intensities may lie in the differential modification of stretches of chromatin. Lopez-Rubio and colleagues demonstrated that the intensity of acetylation/methylation vary in ranges also observed herein [8]. Also, the overall occupancy of histones may vary between loci and along different ORFs as shown by Westenberger and colleagues [20].

With the exception of PFD0070c, all active loci monitored in our study showed clearly higher transcript quantities in schizont stage than in trophozoites. This is in accordance with the study from Wang and colleagues [6] but in contrast to the early work of Kyes and colleagues using the A4 strain [22]. Interestingly, Wang and colleagues found also transcripts from the subtelomeric type B *rif* PFI0025c and the centromeric A2 type *rif* PFD0070c, and also a predominance of PFI0025c transcripts in sporozoites and gametocytes [23]. PFI0025c is probably not exposed at the IRBC surface [24] and probably has a specific, yet unknown role in these parasite forms. Our data suggest that both loci PFI0025c and PFD0070c may also be regulated epigenetically at the studied time points in sporozoites and gametocytes. Our experiments did not show any connection of *rif* transcription and cytoadherence patterns, since the transcripts found in the re-selected parasite line showed transcripts differing from those identified in our previous study (Figure 2 and [5]). This view is also supported by a recently published microarray-based analysis [25]. We could also not identify any connection between the genomic context, transcribed *var* genes and *rif* transcription, at least in the samples from the experiment shown in Figure 2. The dominantly transcribed *var* gene (PFD0615c) is distant from transcribed *rif* genes and no specific pattern of transcript quantity and genomic context (*rif* target located inside a string of *rif* genes or in proximity to *var* genes, see Figure S2 B) was identified.

Lopez-Rubio and colleagues showed that *var* genes that are transcribed after the following reinvasion are marked with H3K4me2 in schizonts when *var* transcription has ceased [12]. In our experiments, loci with low or undetectable transcription showed few modification of this type, however, little modification seemed not predictive for absence of transcriptional activity in the same cycle. Even with a very low level of the “poised mark” modification, transcription may occur (see PFB0040c). We propose that for stronger “poised” modifications in trophozoites, higher transcript quantities are to be expected in schizonts in the same cycle, also coinciding with a higher H3K9 acetylation or low H3K9 trimethylation modification in schizonts. A significant argument against a general rule for the link of active transcription/acetylation at H3K9 and dimethylation at H3K4 is seen at the locus PFD0070c (compare Fig. 1 and Fig. 3), indicating that other factors or histone modifications may have decisive influence on the transcriptional status of a locus, including perhaps the recently discovered modifications at H4 and H4K8 [18]. In any case, more biological replicates would be necessary to clarify the role of the H3K4me2 and possibly the modifications at H4 in the “poised” status of *rif* genes. Upon its first publication in 2007, the poised mark was only tested for the var2csa locus [12].

During the blood stage replication of *P. falciparum*, the parasite must precisely balance epigenetic memory and transcriptional switching in order to optimize its immune evasion through antigenic variation. Although a number of transcriptional regulators were annotated in the *P. falciparum* and some of them were already localized to the nucleus [26], [27] or even shown to associate to silenced chromatin such as HP1 [28], PfKMT1 [8], or to *var* promoter elements [29], the exact events that orchestrate the dynamics of antigenic variation and allelic exclusion are not yet resolved.

Our data demonstrate for the first time that active *rif* loci are marked not only with the H3K9 acetylation mark, but also the poised mark H3K4me2. Also, we showed that silenced loci may either associate to H3K9me3 modified loci or unmodified loci, at least in the stages that were looked at. It is possible that unmodified H3K9 residues are associated to an almost permanently silent locus while H3K9me3 loci were recently switched off or may become switched on. Further, the turnover dynamics are probably faster [5] than for *var* loci. Additional studies targeting the exact chromatin modifications at other histone residues [30] or at H4 [18] comparing active or silent variant gene loci are clearly necessary in order to elucidate the mechanics of recruiting transcription factors to the respective sites. The knowledge of these mechanisms may open new avenues of treating malaria disease by actively interfering or aborting the important virulence mechanism of antigenic variation.

## Materials and Methods

### *P. falciparum* culture, RNA extraction and cDNA preparation

*P. falciparum* strain 3D7 was cultured at 5% hematocrit in RPMI 1640 medium, 10% human plasma. Cultures were maintained at 37°C in Candle jars as described [31]. Cultured parasites were synchronized by intermittent Plasmagel floating [32] followed by Sorbitol synchronization [33] and harvested in middle trophozoite stage (20–24 h post-invasion) or early schizont stage (30–36 h). An aliquot of parasites cytoadherence-selected over Chinese hamster ovary cells expressing CD36 [14] was grown for 10 and 20 generations for ChIP and transcription analysis (Experiment in Figure 1). The 20 generation-parasite line was either grown for additional 5 cycles and tested for chromatin modifications in one blood stage cycle (Experiment in Figure 3) or re-selected twice over CHO-CD36 cells and analyzed for transcription and chromatin modifications (Experiment in Figure 2 and 4).

RNA was extracted using TRIZOL LS (Invitrogen) following instructions of the manufacturer. The washed RNA pellet was briefly dried at RT and dissolved in water and stored at −80°C until use. About 5 µg of total RNA was used for cDNA synthesis. Briefly, total RNA was treated 3 times with DNAse I (Fermentas) prior to synthesis to prevent genomic DNA contamination. The treated RNA was reversed transcribed using MuLV-Reverted aid transcriptase reverse (Fermentas) and random hexamer primers following the manufacturers' instructions.

### Chromatin Immunoprecipitation (ChIP)

ChIP was conducted as described in Methods in Malaria Research [34] with slight modifications. Briefly, chromatin histones from 10^9^ parasites were crosslinked with 1% formaldehyde and RBC were lysed in PBS supplemented with saponin 0.1%. Following saponin lysis, parasites were resuspended in lysis buffer (10 mM Hepes pH 7.9, 10 mM KCl, 0.1 mM EDTA pH 8, 0.1 mM EGTA pH 8, 1 mM DTT), supplemented with 1× Protease Inhibitor Cocktail (Complete, Roche). After 30 minutes of incubation, parasites were lysed using a Dounce Homogenisator (100 strokes), centrifuged for 10 minutes at 14.000 rpm/4°C, and then sonicated using a cell disruptor (Unique Ultrasonic) until resulting DNA fragments had 200–500 bp. At this stage, 10% of the material (“input”) was retrieved and submitted directly to the reversion of crosslinking (see below). For immunoprecipitations, sonicated chromatin was diluted in ChIP buffer (0.01% SDS, 1.1% Triton X-100, 1.2 mM EDTA pH 8, 16.7 mM TrisCl pH 8.1, 150 mM NaCl). Chromatin diluted in ChIP buffer was pre-cleared in 50 µL/mL of a DNA sperm salmon/protein A-sepharose slurry for 2 h/4°C. Afterwards, chromatin was incubated with 1∶250 of anti-H3K9ac, H3K9me_3_, H3K4me_2_ (Upstate) and H3 (Abcam). For negative controls (mock samples) we used an anti-IgG mouse, and for calibration of the overall chromatin quantity, similar amounts of material from the “input” fraction were employed. The chromatin-immune complexes were then washed under stringent conditions. Following this, crosslink was reverted by incubation at 65°C for 4 h and 0.2 M NaCl. DNA was extracted twice with Phenol/Chloroform/isoamylalcohol, precipitated and resuspended in 20–50 µl sterile water. The results presented are the average of two or three independent immunoprecipitations from different extracts of parasites (biological replicates). The significance of differences between signals was evaluated by Student's T test (two tailed) using GraphPad Prism 5.

### Oligonucleotides and qRT-PCR

Oligonucleotides were designed and qPCR was performed as described previously [5]. Briefly, the oligonucleotides were created using the primer3 software based on a fasta-file containing all *rif* transcript sequences and only oligos with perfect primer-to-target specificity were considered. Later, oligonucleotide pairs were selected for global target specificity by ePCR using the 3D7 genome sequence as template. Importantly, the primer performance in qPCRs differed no more than 1 Ct between oligonucleotide pairs used herein and the internal control oligonucleotide pair. PCRs were performed in triplicates and equal amounts of input DNA (0.05–0.5 µg) per imunoprecipitated sample and mock immunoprecipitations (unrelated IgG) were analyzed together with samples. Mock samples presented CTs>35, which was considered as unamplified targets. All *rif* oligos in each immunoprecipitation were calibrated with the input DNA signal using the formula 2^−ΔCt = (CtoligoAb−Ctoligoinput)^, where Ab is the respective antibody used for ChIP. The plotted enrichment was calculated as the ratio of immunoprecipitated Ab oligo signal/immunoprecipitated H3 oligo signal. The internal control transcript used for calibration throughout the experiments was locus seryl t-RNA transferase (PlasmoDB No. PF07.0073), previously shown as reliable (e.g. [21], [35]) since its transcription does not vary significantly during the intraerythrocytic cycle [36]. In some cases, the ratio H3K9ac/H3K9me3 was computed using the values calculated as above. In other cases, we plotted the transcription data against the ChIP data using scatter graphs and calculated the significance of correlation using Pearson's correlation function (one sided probability) in MS Excel.

## Supporting Information

Figure S1 **Ratios of H3K9 modifications plotted against transcript quantities in individual experiments.** Chromatin enrichment and transcript quantification was performed as described in Methods. The relative transcript quantities were plotted against the H3K9ac/me3 signal ratios. All ChIP results are from biological triplicates. In the graphs, Pearson's R^2^ is shown and the corresponding one tailed probability of correlation. The data were calculated using MS Excel. Results for A: material from trophozoites 20–24 h p.i. and 10 reinvasions after panning over CHO-CD36 cells and B, after 20 reinvasions. C: material from trophozoites 20–24 h p.i. (outgrown from thawed cryostabilates of the 20 reinvasion sample) and D, schizonts 30–36 h p.i. from the same reinvasion cycle as in D. E: Material from trophozoites 20–24 h p.i. freshly repanned over CHO-CD36.
(TIF)

Figure S2 **Position of oligos used in qPCR and ChIP in the deduced transcript sequence of analyzed** ***rif*** **targets and their genomic context.** A) The black bars above and the numbers below the scheme of each *rif* coding region indicate the initial position of the amplification of the primer. The numbers at the end of each gene indicate the size of the respective coding regions. The genes were divided in the figure according to the *rif* promoter classification proposed by Joannin et al 2008 [37]. The letters **C** and **T** means telomeric and centromeric position, respectively. *Rif* A type genes are believed to encode IRBC-surface displayed RIFINs while type B RIFINs are probably localized to Maurer's clefts [24]. B) Chromosomal context of the analyzed *rif* loci, extracted from http://plasmodb.org (6/15/2011).
(PPTX)

Figure S3 **Relative quantities of** ***rif*** **transcripts and chromatin enrichment for H3K9ac, H3K9me3 and H3K4me2 (poised mark) in trophozoite and schizont stages for selected** ***rif*** **loci.** A: relative transcript amounts of chosen *rif* targets in trophozoites and schizonts in 3D7 parasites of the same reinvasion, calculated as above. Chromatin enrichment of H3K9ac and H3K9me3 for trophozoites (B) and schizonts (C). The chromatin enrichment was normalized using the qPCR data from ChIP with anti-H3 and 10% of the input. Significant differences between H3K9 trimethylation and acetylation are depicted by single asterisks, and significant H3K4me2 modification (compared to the H3K9me3 control) are indicated by two asterisks (Student's T test, p<0.05).
(TIF)

Figure S4 **Raw results from experiment in** **Figure 4** **showing the influence of primer localization on the qPCR signal after ChIP at two** ***rif*** **loci.** Immunoprecipitated material from the experiment in Fig. 2 was used. The differences between the H3K9ac and H3K9me3 signals (A and B) are not significant at the 95% level (Student's T test, two tailed).
(PPTX)
