# Supplementary figures and images for: Dynamic Activation and Repression of the Plasmodium falciparum rif Gene Family and Their Relation to Chromatin Modification
Source: PLoS One. 2012 Jan 3;7(1):e29881. doi: 10.1371/journal.pone.0029881 (PMC3250495; doi:10.1371/journal.pone.0029881)

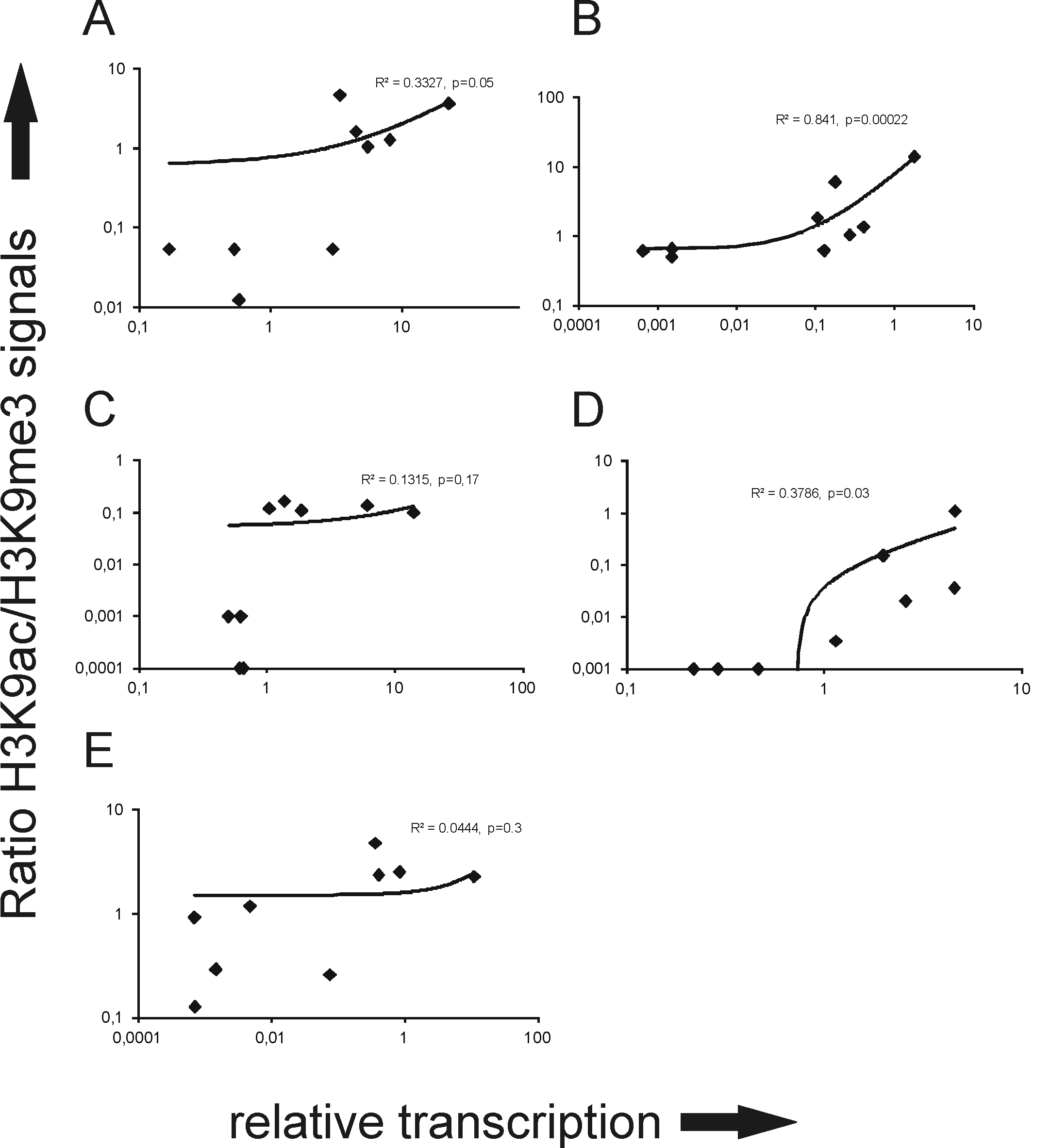

Supplement: Figure S1 — Ratios of H3K9 modifications plotted against transcript quantities in individual experiments. Chromatin enrichment and transcript quantification was performed as described in Methods. The relative transcript quantities were plotted against the H3K9ac/me3 signal ratios. All ChIP results are from biological triplicates. In the graphs, Pearson's R2 is shown and the corresponding one tailed probability of correlation. The data were calculated using MS Excel. Results for A: material from trophozoites 20–24 h p.i. and 10 reinvasions after panning over CHO-CD36 cells and B, after 20 reinvasions. C: material from trophozoites 20–24 h p.i. (outgrown from thawed cryostabilates of the 20 reinvasion sample) and D, schizonts 30–36 h p.i. from the same reinvasion cycle as in D. E: Material from trophozoites 20–24 h p.i. freshly repanned over CHO-CD36. (TIF) [file pone.0029881.s001.tif]

## Slide 1
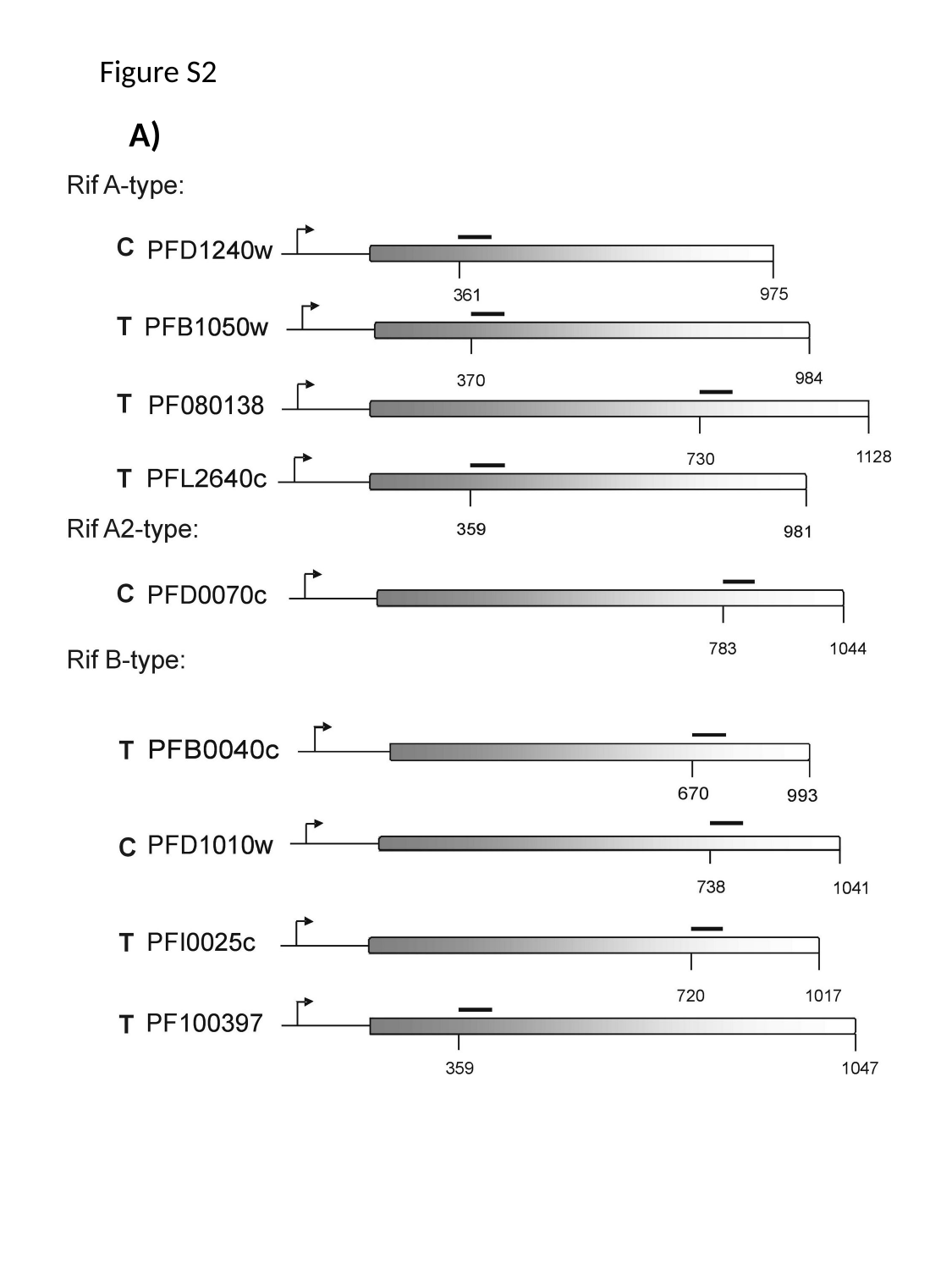

Figure S2
A)

## Slide 2
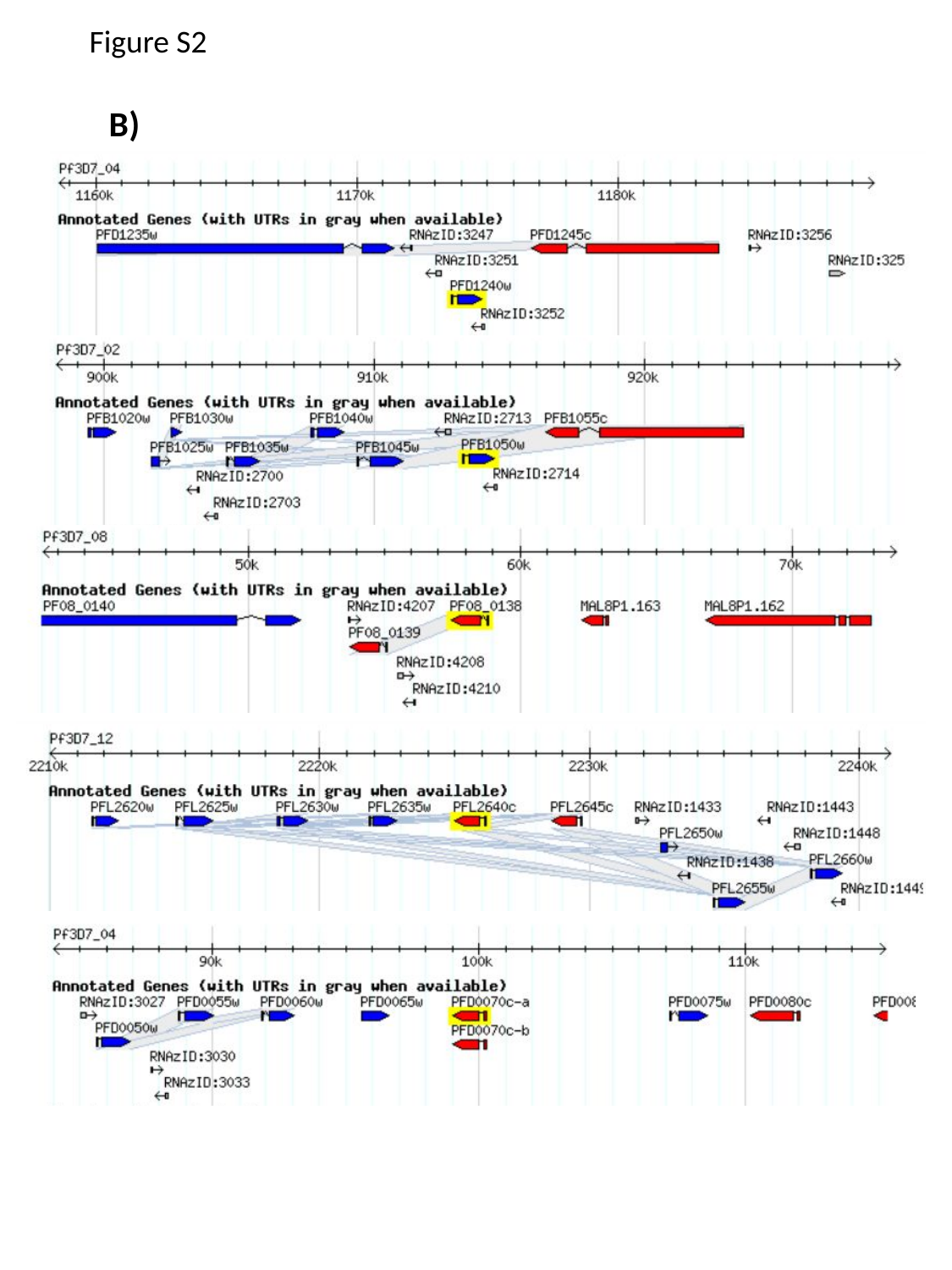

Figure S2
B)

## Slide 3
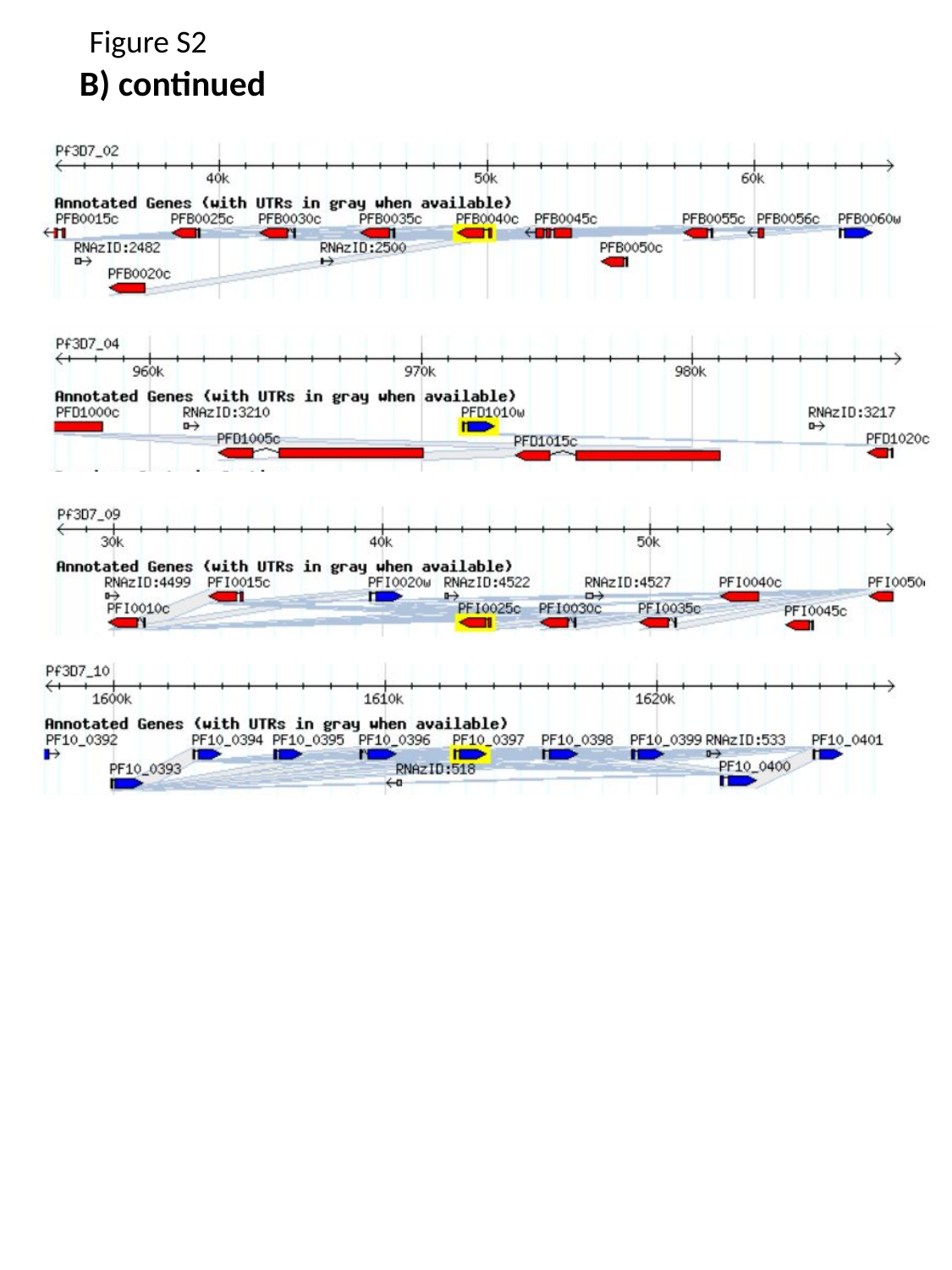

Figure S2
B) continued

Supplement: Figure S2 — Position of oligos used in qPCR and ChIP in the deduced transcript sequence of analyzed rif targets and their genomic context. A) The black bars above and the numbers below the scheme of each rif coding region indicate the initial position of the amplification of the primer. The numbers at the end of each gene indicate the size of the respective coding regions. The genes were divided in the figure according to the rif promoter classification proposed by Joannin et al 2008 [37]. The letters C and T means telomeric and centromeric position, respectively. Rif A type genes are believed to encode IRBC-surface displayed RIFINs while type B RIFINs are probably localized to Maurer's clefts [24]. B) Chromosomal context of the analyzed rif loci, extracted from http://plasmodb.org (6/15/2011). (PPTX) [file pone.0029881.s002.pptx]

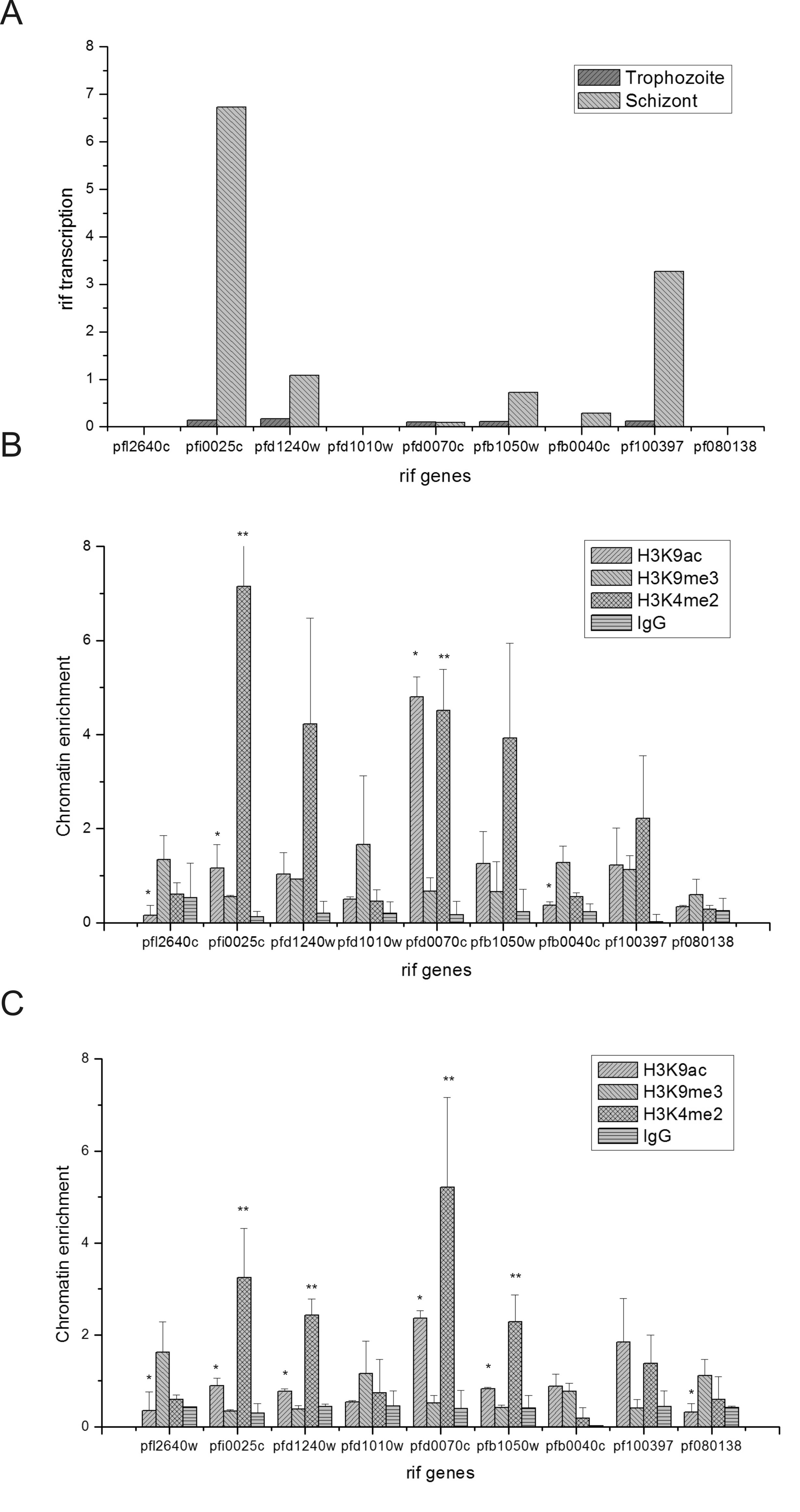

Supplement: Figure S3 — Relative quantities of rif transcripts and chromatin enrichment for H3K9ac, H3K9me3 and H3K4me2 (poised mark) in trophozoite and schizont stages for selected rif loci. A: relative transcript amounts of chosen rif targets in trophozoites and schizonts in 3D7 parasites of the same reinvasion, calculated as above. Chromatin enrichment of H3K9ac and H3K9me3 for trophozoites (B) and schizonts (C). The chromatin enrichment was normalized using the qPCR data from ChIP with anti-H3 and 10% of the input. Significant differences between H3K9 trimethylation and acetylation are depicted by single asterisks, and significant H3K4me2 modification (compared to the H3K9me3 control) are indicated by two asterisks (Student's T test, p<0.05). (TIF) [file pone.0029881.s003.tif]

## Slide 1
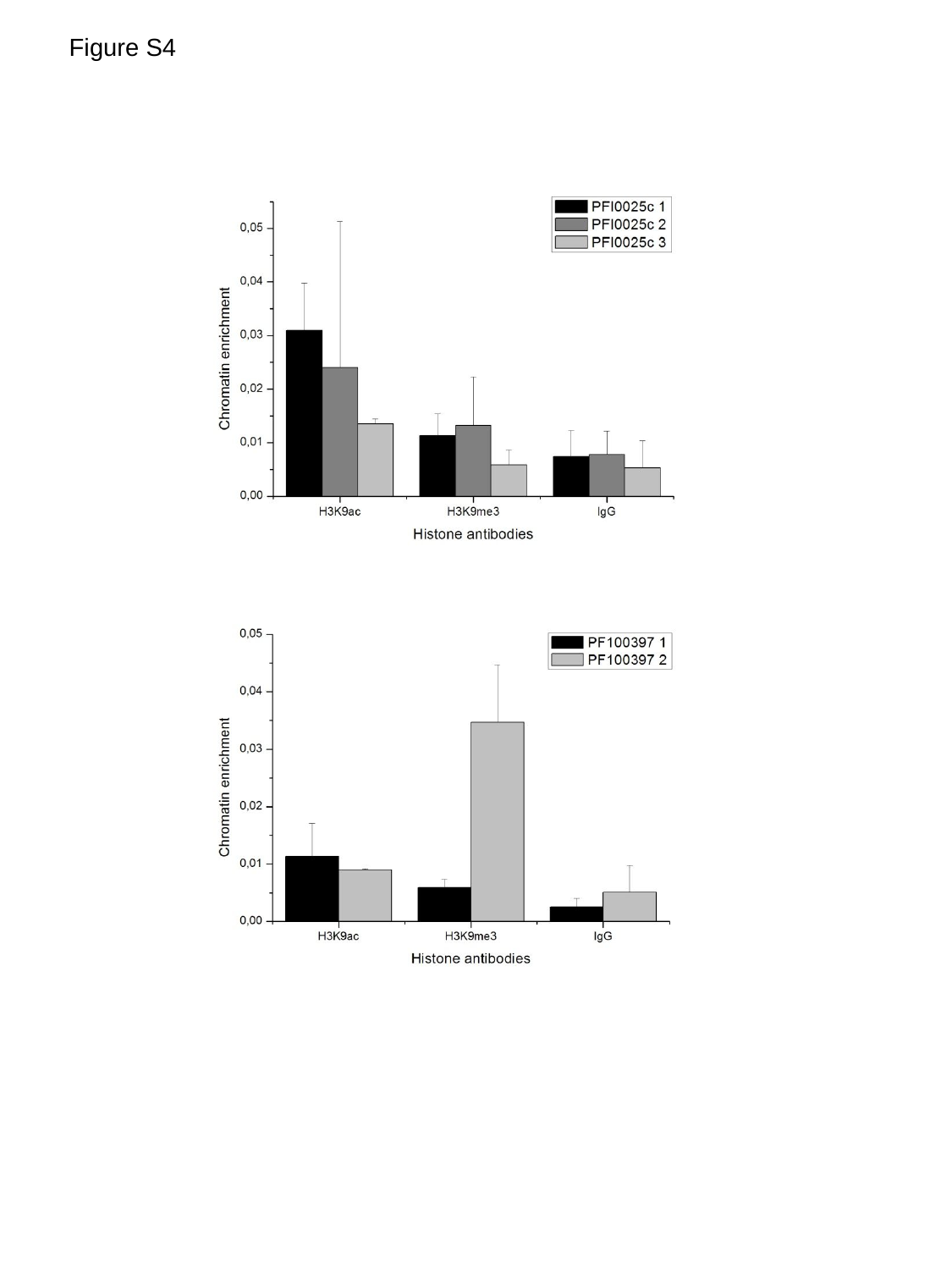

Figure S4

Supplement: Figure S4 — Raw results from experiment in Figure 4 showing the influence of primer localization on the qPCR signal after ChIP at two rif loci. Immunoprecipitated material from the experiment in Fig. 2 was used. The differences between the H3K9ac and H3K9me3 signals (A and B) are not significant at the 95% level (Student's T test, two tailed). (PPTX) [file pone.0029881.s004.pptx]
